# Evaluating the association between *MPDZ-NF1B* rs1324183 and keratoconus in an independent northwestern Chinese population

**DOI:** 10.1186/s12886-022-02359-1

**Published:** 2022-03-19

**Authors:** Shiqin Yuan, Dong Li, Meijiao Ma, Lingjie Zhou, Zhen Ma, Baoyu Shi, Shuang Zhang, Huiping Li, Xunlun Sheng, Junxiu Liu

**Affiliations:** 1 Ningxia Clinical Research Center of Blinding Eye Disease, People Hospital of Ningxia Hui Autonomous Region (People’s Hospital of Autonomous Region Affiliated to Ningxia Medical University), No 936, Huanghe East Road, Jinfeng District, Yinchuan, 750001 Ningxia China; 2 The Affiliated Xuzhou Municipal Hospital of Xuzhou Medical University, Xuzhou First People’s Hospital, No 269, University Road, Tongshan District, Xuzhou, 221116 Jiangsu China; 3 Gansu Aier Ophthalmology and Optometry Hospital, 1228-437, Guazhou Road, Qilihe District, Lanzhou, 730050 Gansu China

**Keywords:** Keratoconus, rs1324183 (*MPDZ-NF1B*), Association, Genetic mark, Corneal parameter

## Abstract

**Background:**

Keratoconus (KC) is a complex, non-inflammatory corneal degenerative disease. Although numerous studies have analyzed the correlation of SNP rs1324183, which located in *MPDZ-NF1B* gene, and KC in different populations, only few findings were repeated. In this study, to evaluate the association between rs1324183 and KC in a new independent Chinese population, we performed a replication study of the significantly associated rs1324183.

**Methods:**

In total of 114 unrelated KC patients and 88 unrelated controls were recruited from Ningxia, China. We detected the genotypes and alleles of rs1324183 using PCR technology and Sanger sequencing and also analyzed the association between this locus and KC, its clinical parameters by statistical methods.

**Results:**

The frequency of genotype AA (11, 9.6%) and genotypes containing allele A (47, 41.2%) of rs1324183 in KC were both higher than those of the control group. And genotype AA of rs1324183 conferred a higher risk of KC (OR > 1). Moreover, corneal parameter Belin/Ambrósio enhanced ectasia display final D value (BAD-D) had significant correlation (*p* = 0.002) with AA genotype of rs1324183 in KC.

**Conclusions:**

Our replication study indicates that the results of rs1324183 associated with KC in our population is robust and further better illustrates the significance of BAD-D as a diagnostic indicator for KC. rs1324183 should be considered as the first genetic mark of KC risk in its future diagnosis.

**Supplementary Information:**

The online version contains supplementary material available at 10.1186/s12886-022-02359-1.

## Background

Keratoconus (KC) is a complex, non-inflammatory corneal degenerative disease characterized by corneal dilatation, cone-shaped protrusion, irregular myopia, and astigmatism, which usually affects on both eyes, but the progress of the eyes is asymmetrical [1–3]. KC occurs more often in adolescence, and severe visual impairment occurs with the thinning of the corneal stromal layer of cornea. It still remains one of the most frequent causes of corneal transplantation [4–6].

Researchers have gradually discovered that KC is no longer a rare disease. Some areas of the world, due to genetic and environmental factors, are suffering from an epidemic outbreak of KC. A review of early studies of the prevalence of KC found a range of 50–230 cases per 100,000 (0.05–0.23%) populations in Western countries [3]. However, several recent independent studies have found the prevalence to be higher. In a India study, they estimated it to be 2.3% in general population (and higher in refractive surgery cases) [7, 8]. A longitudinal cohort study from Australia found the prevalence to be as high as 1.2% among 20-year-olds [9]. The first study on population-based KC prevalence in a randomized sample in Turkey showed 2.4% (2393/100000) [10]. While a systematic Review and Meta-Analysis from 15 countries calculated a prevalence of 0.13% in the whole population [11]. Therefore, estimates for the prevalence of keratoconus have varied and it also has not been accurately determined. Different ethnicity living in the different geographic location suggests a genetic basis as well as environmental impact of disease. For example, higher prevalence than the European average has been found in Asian [12–20]. According to two genome-wide association studies of keratoconus, as of March 2021, approximately 66 variants from 50 genes were identified to be significantly associated with KC in different populations [21, 22]. However, the relationship between these variants and the pathogenesis of keratoconus is still unclear. Especially, whether the variants can really affect the occurrence of keratoconus and can be used as genetic markers for the early diagnosis of keratoconus remain to be further studied.

In recently years, Wang YM and Hao XD et al. both analyzed the association of SNP locus with keratoconus in Chinese population, and revealed that the rs1324183 (*MPDZ-NF1B*), which located between the *MPDZ* and *NF1B* genes, is a putative genetic marker for monitoring the progression of keratoconus and should be further investigated in other different Chinese populations [23, 24]. Thence, in this study, we collected a total of 114 KC patients who live in northwestern of China for the first time and further confirmed the association between the rs1324183 (*MPDZ-NF1B*) and KC, providing a clear basis for early clinical diagnosis in Chinese population.

## Methods

### Subjects

A total of 114 sporadic Chinese KC patients and 88 unrelated controls from Ningxia Hui Autonomous region were recruited from the Ningxia Eye Hospital from Nov. 2017 to Nov.2019. The diagnostic criteria of KC is based on the consensus on the diagnosis of keratoconus in China, which take in and learn from the international grading method of keratoconus [25–28] as well as grading method of keratoconus of Lixin Xie and Weiyun Shi [29]. Patients with keratoconus have a history of myopia and astigmatism, and the best corrected visual acuity (BCVA) is less than 1.0. At least one of the slit lamp microscopy will be positive, such as corneal stroma thinning, tapered lordosis, Fleischer ring, Vogt’s striae, epithelial or subepithelial scar. The corneal topography shows that the central diopter of the anterior surface of the cornea is greater than 47.00 D. Therefore, the consensus on the diagnosis of keratoconus in China is divided into 4 stages of incubation period, initial period, completion period, and scarring period. The corneal topography of the affected eye during the incubation period is normal, the corrected visual acuity is greater than or equal to 1.0. The initial patient’s best corrected visual acuity (BCVA) is greater than or equal to 0.8, but the corneal topography examination has keratoconus manifestations (central diopter of the anterior surface of the cornea is greater than 47 D). The BCVA of the affected eye during the completion period is less than 0.8, and they have at least one typical clinical symptom of KC, such as corneal stroma thinning, tapered lordosis, Fleischer ring, and Vogt striae. The affected eye in the scarring period have residual scar on the entire cornea after the acute keratoconus edema subsiding.

In this study, each KC patient has at least one eye with typical clinical characteristics from the completion period, with a BCVA < 0.8. The study protocol was approved and reviewed by the Ethics Committee on Human Research at People Hospital of Ningxia Hui Autonomous Region. Written informed consent was received from each participant or his/her legal guardians before participation, and study adhered to the tenets of the declaration of Helsinki.

All standard ophthalmic examination of participants were performed by comprehensive refractometry (VT-10, TOPCON, Japan), slit-lamp biomicroscopy (Topcon, Tokyo, Japan), intraocular pressure (IOP), anterior segment analysis system (Pentacam 70,700, Germany), and corneal biomechanics analyzer (Corvis ST 72,100, Germany). Slit lamp biomicroscopy was used to identify stromal corneal thinning, Vogt’s striae, or a Fleischer ring. Best-corrected visual acuity (BCVA) was performed by comprehensive optometry. Anterior segment analysis system measured 9 corneal parameters of the thinnest point thickness (TP), max keratometry (K max), Belin/Ambrósio enhanced ectasia display final D value (BAD-D), deviation of normality of the front elevation (Df), deviation of normality of the back elevation (Db), deviation of normality of pachymetric progression (Dp), deviation of normality of corneal thinnest point (Dt), deviation of normality of relational thickness (Da), and Ambrósio’s relational thickness (ARTH). And 6 other corneal parameters of corvis biomechanical index (CBI), deformation amplitude ratio (Da_Ratio), adjusted AP1-bIOP (SPA1), biomechanical intra occular pressure (bIOP), integrated radius, and tomographic and biomechanical index (TBI) were recorded by corneal biomechanics analyzer. All results of corneal parameters will generate the corresponding quality factor (QS). When QS > 95%, an “OK” display appears on the test instrument, indicating that the quality of the test data is acceptable. If the test quality is not good enough, they must be retested. In order to avoid detection errors, the operations mentioned above should be effectively checked by the same experienced medical technician for at least three times. The higher quality results will be selected into the test group.

The control subjects were recruited from the patients who were scheduled to undergo laser surgery for myopia and volunteers. Among them, only one eye was randomly selected for analysis in each control group. Except for astigmatism and myopia, there are no any other ophthalmological diseases. The corneal topography examination of both eyes were normal, and the best corrected visual acuity were great than or equal to 1.0.

### rs1324183 (*MPDZ-NF1B*) detection and genotyping

rs1324183 in *MPDZ-NF1B* was reported to be associated with keratoconus [23, 24, 30, 31]. Genomic DNA was extracted from peripheral blood of all participants using the QIAamp DNA Mini Kit (Qiagen, Germany) according to the manufacturer’s protocol. DNA concentration was inspected by Nanodrop 2000 (Thermo Fisher, USA). The target sequence which contained the rs1324183 site (Primers, forward: 5’-TCCTACCAGCTTGTCTCCAAA-3’, reverse: 5’-ACAAGAAGCCACAAGTCTGGC-3’) were amplified by ordinary PCR (Allsheng, Hangzhou, China) and then conducted Sanger sequencing on ABI 3730 analyzer (Applied, Biosystem). Sites of variation were identified through a comparison of DNA sequences with the corresponding GenBank reference sequences using the Mutation Surveyor software, version 5.0.0. Genotyping (Heterozygous mutation, homozygous mutation and wild-type) were classified based on the results of Sanger sequencing.

### Statistical analysis

All statistical analyses were performed using SPSS 22.0 (IBM Corporation, Armonk). Statistical significance was declared at a = 0.05. Kolmogorov–Smirnov test (K-S test) and the Levene test were used respectively to test whether the data conformed to the normal distribution and to evaluate the homogeneity of variance of the data between groups. The Hardy–Weinberg equilibrium (HWE) *p* value and minor allele frequency (MAF) were estimated using Haploview software, version 4.2 (http://www.broad.mit.edu/mpg/haploview/). We also assessed the odds risk (OR) of susceptibility locus for keratoconus carrying specific genotypes based on the binomial Logistic regression analysis. In order to investigate the most sensitive and specific indicators of Pentacam and Corvis ST in the diagnosis of keratoconus, we drew the receiver operating characteristic curve (ROC) of corneal parameters and then compared the areas under ROC (AUC) to obtain the best diagnostic parameters of KC. If the AUC is between 0.5 and 0.7, the diagnosis efficiency is low. An area between 0.7–0.9 indicates medium diagnostic efficiency. More than 0.9 indicates high diagnostic efficiency. Moreover, in keratoconus group, variance trend analysis was used to assess the difference of average values of 15 corneal parameters (K max, BAD-D, Df, Db, Dp, Dt, Da, ARTH, CBI, Da_Ratio, bIOP, TP, integrated radius, TBI, and SPA1) in different genotypes of rs1324183 (*MPDZ-NF1B*).

## Results

### Association of gender, age, and *MPDZ-NF1B* rs1324183 with keratoconus

**Fig. 1:**
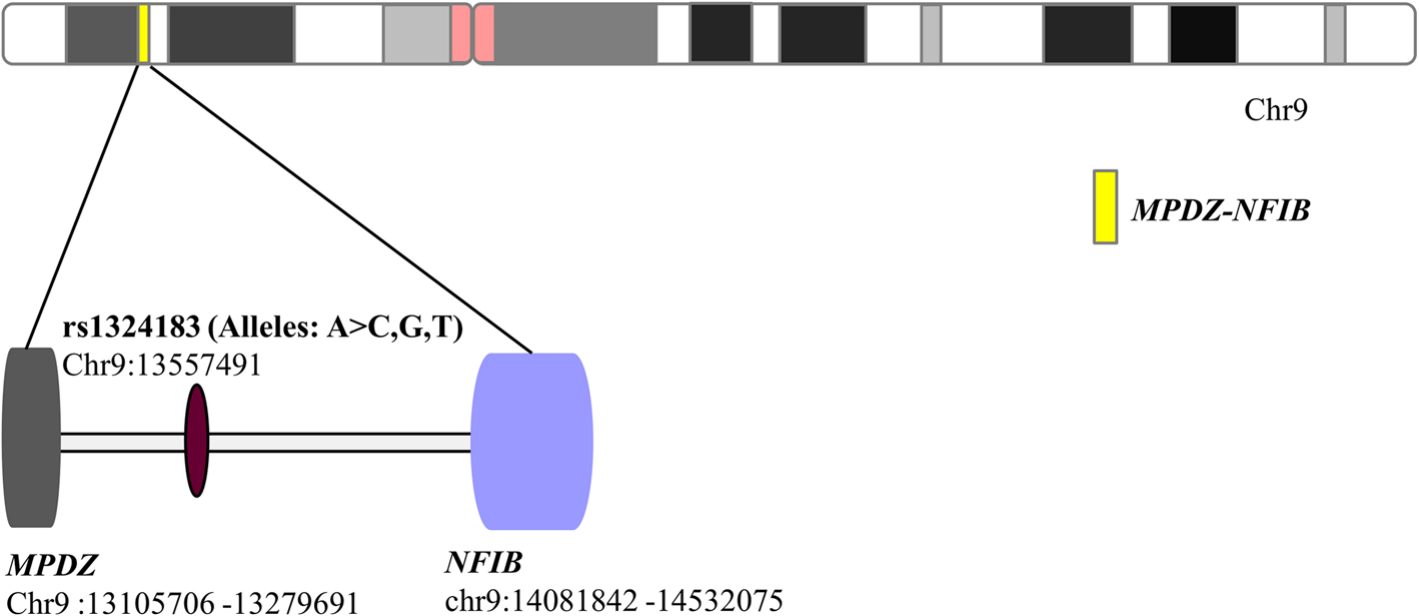
Localization of *MPDZ-NFIB* rs1324183 in the chromosome 9. rs1324183 locates in the intergenic region of *MPDZ* and *NFIB* in the 9p23 band of chromosome 9. It has four alleles in this gene locus, alleles: A > C,G,T. A and C are the most common alleles, while G and T are less common

The rs1324183, which locates in the intergenic region of *MPDZ* and *NFIB*, is shown in Fig. 1. Hardy–Weinberg equilibrium *p* values and the minor allele frequency of the studied SNP (rs1324183) are shown in Table 1. rs1324183 was in Hardy–Weinberg equilibrium in both patient and control groups, respectively. And they all had a MAF greater than 0.05 in our population. Besides, Basic characteristics of our participants were further analyzed in Table 1, and we found no significant differences in gender and age between KC cases and controls. However, the genotype distributions of rs1324183 (*MPDZ-NFIB*) (*p* = 0.032) were significantly different between the patients and controls (Table 2). Especially, the frequency of genotype AA (11, 9.6%) and genotypes containing allele A (47, 41.2%) of rs1324183 in KC were both higher than those of the control group. Genotype AA of rs1324183 (*MPDZ-NFIB*) had an OR of 3.475 (95% CI = 1.241–8.431, *p* = 0.003) under the recessive model and an OR of 2.108 (95% CI = 0.464–1.085, *p* = 0.001) under the dominant model (Table 2). These results confirmed that the genotype AA of rs1324183 (*MPDZ-NFIB*) was an important KC risk genotype in our population.

**Table 1:** Basic characteristics of KC patients and controls

**Factors**	**Chr**	**Locus**			**Patients (*n* = 114)**		**Controls (*n* = 88)**		
				**Minor**	**HWE**		**HWE**		
			**Alleles**	**allele**	***p* value**	**MAF**	***p* value**	**MAF**	***p* value**
SNP rs1324183	9	*MPDZ-NF1B*	A/C	A	0.076	0.119	0.074	0.254	
Sex (%)					**M**	**F**	**M**	**F**	
					59(51.8%)	55(47.8%)	49(55.7%)	39(44.3%)	0.34
Age (mean ± SD)					25.12 ± 10.34		25.58 ± 9.63		0.74

*SNP* single-nucleotide polymorphism, *HWE* Hardy–Weinberg equilibrium, *MAF* minor allele frequency, *M* Male, *F* Female, *SD* standard deviation

**Table 2:** Genotype frequencies of studied SNP and their association with susceptibility to KC

						**Recessive model**		**Dominant model**	
**SNP/Group**	**Genotype frequency, n (%)**				***p***	**OR (95%Cl)**	***p***	**OR (95%Cl)**	***p***
rs1324183	AA + CA	AA	CA	CC		AA versus CA&CC		AA &CA versus CC	
Control	18(20.5)	3(3.4)	15(17.1)	70(79.5)					
Patients	47(41.2)	11(9.6)	36(31.6)	67(58.8)	0.032	3.475(1.241–8.431)	0.003	2.108(0.464–1.085)	0.001

The risk genotype of rs1324183 (*MPDZ-NF1B*) was the genotype AA

### The diagnostic efficacy of corneal parameters for keratoconus

**Fig. 2:**
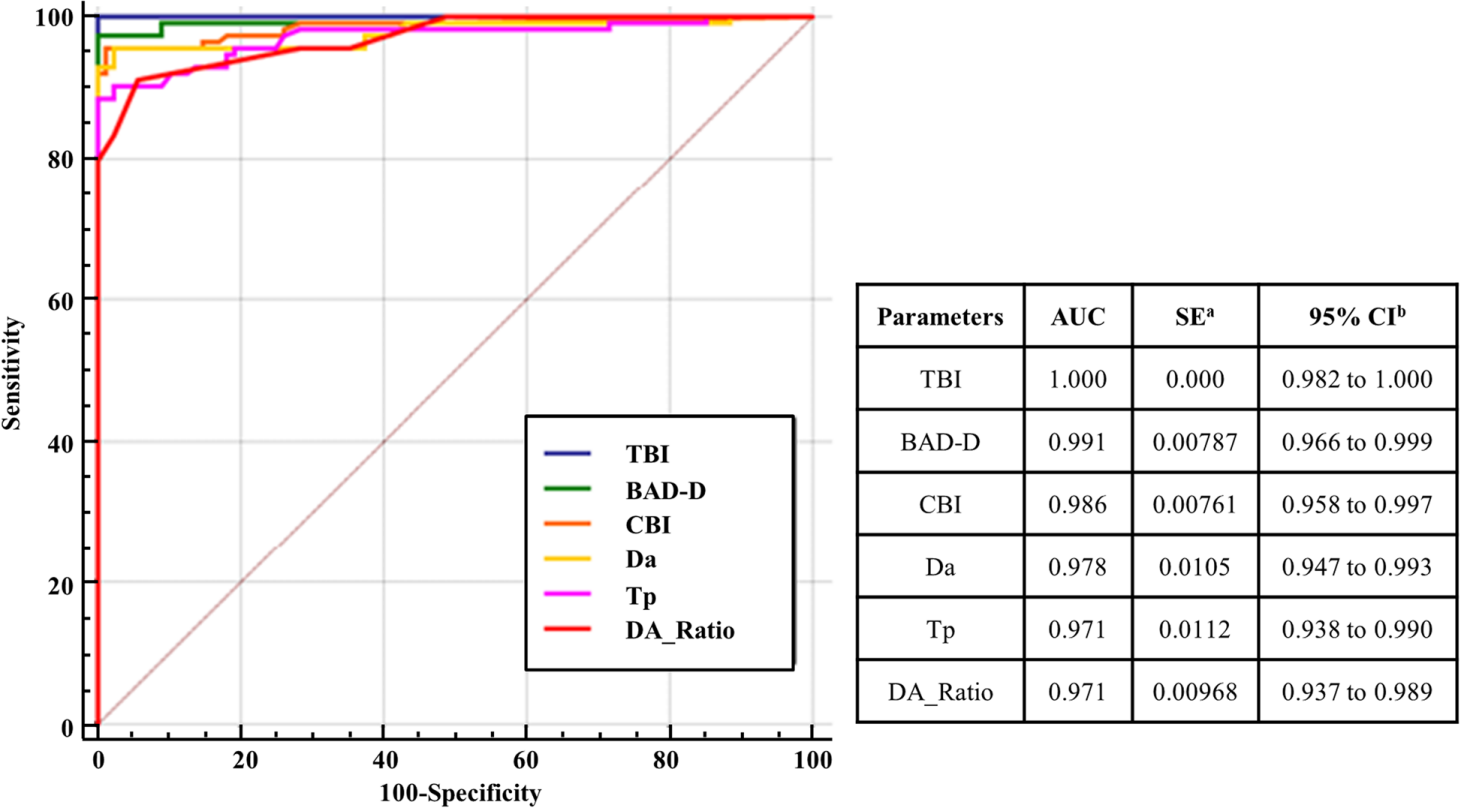
Comparison of the area under receiver operating characteristic curve (AUC) of the first six corneal parameters. Receiver operating characteristic curve (ROC) of TBI, BAD-D, CBI, Da, Tp, and Da_Ratio. Blue line represents TBI, Green line represents BAD-D, Orange line represents CBI, Yellow line represents Da, Pink line represents Tp, and Red line represents Da_Ratio. TBI, BAD-D, and CBI have larger AUC than the other three parameters

To evaluate the diagnostic efficacy of corneal parameters for keratoconus, we further analyzed the correlation between corneal parameters and keratoconus and found that, compared with the control, the KC cases had relatively higher mean db, dp, df, da, dt, BAD-D, CBI, TBI, K max, integrate radius, and Da_Ratio, while the mean of TP, bIOP, SPA1, and ARTH were relatively lower (Table 3). It suggests that all corneal parameters mentioned above may be indicators of KC diagnosis. Then, we studied the sensitivity and specificity of 15 corneal parameters in the diagnosis of KC and found that all the areas under receiver operating characteristic curves (AUC) of corneal parameters except bIOP were greater than 0.9, indicating that they have a high diagnostic efficiency for KC (Table 4). In addition, we found that the first six AUC of corneal parameters in our population were TBI (AUC = 1.000), BAD-D (AUC = 0.991), CBI (AUC = 0.986), Da (AUC = 0.978), Tp (AUC = 0.971), and Da_Ratio (0.971) (Fig. 2). Although the areas under receiver operating characteristic curves of these six corneal parameters appeared to be greater than 0.9, the youden index and specificity of TBI, BAD-D, and CBI were significantly higher than the other three parameters (Table 4). It suggests that TBI, BAD-D, and CBI are more reliable in the diagnosis of keratoconus.

**Table 3:** Analysis of corneal parameters in keratoconus and control group

Parameters	Controls (*n* = 88)	Keratoconus (*n* = 114)	*p*
	**Mean ± SD**	**Mean ± SD**	
Tp	546.44 ± 27.68	439.61 ± 78.92	< 0.01
BAD-D	1.20 ± 0.46	10.15 ± 14.61	< 0.01
Df	0.11 ± 0.93	10.56 ± 10.10	< 0.01
Db	-0.14 ± 3.05	10.16 ± 17.23	< 0.01
Dp	2.30 ± 8.58	14.49 ± 46.59	< 0.01
Dt	-0.14 ± 0.81	8.89 ± 43.27	< 0.01
Da	0.73 ± 0.56	2.85 ± 0.81	< 0.01
K max	49.13 ± 43.68	55.37 ± 10.94	< 0.01
ARTH	495.12 ± 79.59	218.42 ± 129.14	< 0.01
CBI	0.051 ± 0.086	0.88 ± 0.24	< 0.01
TBI	0.23 ± 0.20	0.99 ± 0.03	< 0.01
Integrated radius	7.46 ± 0.74	11.11 ± 3.78	< 0.01
Da_Ratio	4.13 ± 0.29	5.47 ± 1.03	< 0.01
bIOP	16.69 ± 2.14	14.82 ± 3.53	< 0.01
SPA1	106.46 ± 15.23	69.35 ± 19.46	< 0.01

*AUC* the areas under ROC, *TP* the thinnest point thickness, *K max* max keratometry, *BAD-D* Belin/Ambrósio enhanced ectasia display final D value, *Df* deviation of normality of the front elevation, *Db* deviation of normality of the back elevation, *Dp* deviation of normality of pachymetric progression, *Dt* deviation of normality of corneal thinnest point, *Da* deviation of normality of relational thickness, *ARTH* Ambrósio’s relational thickness, *CBI* corvis biomechanical index, *Da_Ratio* deformation amplitude ratio, *SPA1* adjusted AP1-bIOP, *bIOP* biomechanical intra occular pressure, *TBI* tomographic and biomechanical index

**Table 4:** Analysis of sensitivity and specificity of 15 corneal parameters in the diagnosis of KC

Parameters	**Sensitivity**	**Specificity**	**AUC**	**Youden index**	***p***
Tp	88.6	100	0.971	0.889	< 0.001
Df	88.6	100	0.957	0.886	< 0.001
Db	78.95	97.73	0.925	0.7667	< 0.001
Dp	85.96	97.73	0.921	0.8369	< 0.001
Dt	85.96	100	0.959	0.8596	< 0.001
Da	95.61	97.73	0.978	0.9334	< 0.001
**BAD-D**	**97.37**	**100**	**0.991**	**0.9737**	** < 0.001**
**CBI**	**95.61**	**98.86**	**0.986**	**0.9448**	** < 0.001**
**TBI**	**100**	**100**	**1**	**1**	** < 0.001**
bIOP	59.65	68.18	0.665	0.2783	< 0.001
K max	84.21	98.86	0.952	0.8307	< 0.001
SPA1	92.11	89.77	0.953	0.8188	< 0.001
Integrated radius	92.11	92.05	0.965	0.8415	< 0.001
ARTH	92.98	95.45	0.966	0.8844	< 0.001
Da_Ratio	91.23	94.32	0.971	0.8555	< 0.001

### Correlation between the corneal parameters and rs1324183 (*MPDZ-NFIB*) in keratoconus group

To investigate whether the risk genotype of keratoconus is related to its reliable diagnostic parameters, we analyzed the correlation between the corneal parameters and rs1324183 (*MPDZ-NFIB*) in our KC cases and found that the mean db, dp, da, BAD-D, K max, and integrate radius in KC patients homozygous for the risk allele A at SNP rs1324183 (26.549 ± 34.398 D, 49.091 ± 103.675 D, 3.934 ± 0.451 D, 24.948 ± 29.538 D, 70.636 ± 17.694 D, and 15.609 ± 7.676 D respectively) were significantly higher than patients with the genotype CA (9.961 ± 20.732 D, 16.975 ± 58.199 D, 2.778 ± 0.686 D, 10.022 ± 17.577 D, 54.147 ± 8.427 D, and 10.911 ± 2.718 D, respectively) and CC (7.583 ± 7.044 D, 7.481 ± 6.289D, 2.718 ± 0.794 D, 7.781 ± 5.226 D, 53.521 ± 8.753 D, and 10.479 ± 2.804 D, respectively) (Table 5). In contrast, patients with genotype AA at SNP rs1324183 had relatively lower mean Tp (312.818 ± 118.996 D), bIOP (10.500 ± 5.856 D), SPA1 (45.655 ± 19.401 D), and ARTH (63.664 ± 54.674 D) compared with the patients with CA (447.083 ± 68.893 D, 15.031 ± 3.341 D,69.686 ± 17.856 D, and 221.394 ± 122.248 D, respectively) and CC (456.418 ± 54.807 D, 15.418 ± 2.589 D, 73.057 ± 17.736 D, 242.236 ± 124.917 D) (Table 5). In addition, no other corneal parameters attained significant difference when comparing among different genotype groups. It showed that the KC risk genotype (genotype AA of rs1324183) was significantly associated with 10 specific corneal parameters of db, dp, da, BAD-D, K max, integrate radius, TP, bIOP, SPA1, and ARTH, suggesting that these 10 specific corneal parameters may play an important role in predicting the risk of KC. What’s more, one result actually echoes the findings in Tables 3 and 4, we found that only BAD-D showed a stable and strong association with KC risk in our population, indicating that the BAD-D should be confirmed in more future studies.

**Table 5:** Correlation of rs1324183 (*MPDZ-NF1B*) with corneal parameters in keratoconus

Parameters	Genotype	*N*	Mean	SE	P trend weighted	*p*
Tp	AA	11	312.818	118.996	0.000	0.000
	CA	36	447.083	68.893		
	CC	67	456.418	54.807		
df	AA	11	19.907	16.853	0.036	0.15
	CA	36	9.48	8.483		
	CC	67	9.598	8.795		
db	AA	11	26.549	34.398	0.004	0.02
	CA	36	9.961	20.732		
	CC	67	7.583	7.044		
dp	AA	11	49.091	103.675	0.007	0.008
	CA	36	16.975	58.199		
	CC	67	7.481	6.289		
dt	AA	11	16.81	21.772	0.099	0.225
	CA	36	17.393	75.723		
	CC	67	3.031	2.784		
da	AA	11	3.934	0.451	0.004	0.005
	CA	36	2.778	0.686		
	CC	67	2.718	0.794		
**BAD-D**	**AA**	**11**	**24.948**	**29.538**	**0.002**	**0.002**
	**CA**	**36**	**10.022**	**17.577**		
	**CC**	**67**	**7.781**	**5.226**		
CBI	AA	11	1	0	0.025	0.08
	CA	36	0.926	0.161		
	CC	67	0.844	0.286		
TBI	AA	11	1	0	0.439	0.708
	CA	36	1	0		
	CC	67	1	0.003		
bIOP	AA	11	10.5	5.856	0.005	0.01
	CA	36	15.031	3.341		
	CC	67	15.418	2.589		
K max	AA	11	70.636	17.694	0.003	0.004
	CA	36	54.147	8.427		
	CC	67	53.521	8.753		
SPA1	AA	11	45.655	19.401	0.003	0.007
	CA	36	69.686	17.856		
	CC	67	73.057	17.736		
Integrate radius	AA	11	15.609	7.676	0.015	0.046
	CA	36	10.911	2.718		
	CC	67	10.479	2.804		
ARTH	AA	11	63.664	54.674	0.002	0.005
	CA	36	221.394	122.248		
	CC	67	242.236	124.917		
Da_Ratio	AA	11	6.382	1.264	0.319	0.363
	CA	36	5.278	0.495		
	CC	67	5.425	1.127		

## Discussion

Since the early clinical manifestations of keratoconus are not obvious, and the overlap and low sensitivity of clinically specific indicators often happens in some keratoconus and some normal corneas with abnormal morphology or thin thickness, which has been a difficulty for ophthalmologists in early diagnosis of keratoconus. Although a growing number of researchers have identified some genetic markers associated with keratoconus [21, 23, 24], the reliability of most results has yet to be widely replicated and confirmed.

In this study, we assessed the replication correlation between SNP rs1324183 and keratoconus in our northwestern population of China, which have never been confirmed in such population. SNP rs1324183 (*MPDZ-NFIB,* chr9:13,557,491) was first screened associated with the central corneal thickness (CCT) in an Asian cohort in 2012 [32], and then was studied in different KC population in the world [23, 24, 30, 31, 33]. They found that SNP rs1324183 was significantly associated with KC (p < 0.05), conferring a high risk towards KC, and should be used as genetic markers for the diagnosis of keratoconus in the future. In our study, similarly, we also obtained the close correlation between SNP rs1324183 and KC, in particular, the allele A and the genotype AA of rs1324183 (*MPDZ-NFIB*) were both confirmed as higher risks for keratoconus in our population (Tables 1 and 2). However, we did not find any differences in gender and age between our KC cases and controls (Table 1). Results concerning gender and age preponderance on keratoconus vary between previous studies. Most of them demonstrated that gender and age both associated with high prevalence of KC [34–40], but some people reported that there was no gender preponderance in KC and also no significant association between age and the prevalence of KC [11, 41]. In our study, no difference in gender and age between KC cases and controls could be because of the small number of subjects.

In addition, we also evaluated the diagnostic efficacy of visualized corneal biomechanics analysis system combined with Pentacam related parameters in our KC patients, 15 corneal parameters (Tp, df, db, dp, dt, da, BAD-D, K max, ARTH, CBI, TBI, integrate radius, Da_Ratio, bIOP, and SPA1) of Pentacam and Corvis ST were included and analyzed in the study. According to reports, even if no corneal morphological abnormality is observed in the early stage of keratoconus, it generally exist corneal biologic and biomechanical abnormalities [42, 43]. Therefore, we saw assessing the diagnostic efficacy of corneal biologic and biomechanical parameters for KC as vital. In the present study, we found that all corneal biologic and biomechanical parameters could differentiate the KC patient from the controls in our population (Table 3). But the sensitivity and specificity of their diagnosis for KC were different. Further study showed that the areas under the receiver operating characteristic curve (AUC) as well as the Youden index of TBI, CBI, and BAD-D were greater than those of the other 12 corneal parameters, indicating that TBI, CBI, and BAD-D all have higher diagnostic efficiency for KC (Table 4, Fig. 2). Similar findings on them were also reported by Hashemi and Salomão MQ Jr et al. [44, 45].

Notably, many studies have reported that the SNP rs1324183 (*MPDZ-NFIB*) is a genetic marker highly correlated with KC cases [23, 24, 30, 31, 33], and in our population, we have also further confirmed that the genotype AA of rs1324183 confers a higher risk for KC (Table 2). However, few researchers analyzed the correlation between rs1324183 (*MPDZ-NFIB*) and kinds of corneal biometric and biomechanical parameters in the KC cases. In this study, we explored the correlation between each corneal parameter and rs1324183 (*MPDZ-NFIB*) in KC patients, and found that BAD-D was the best corneal biometric parameter for diagnosing clinical KC cases. As several studies have shown BAD-D to be a strong parameter to differentiate both keratoconus and subclinical keratoconus from normal corneas [46, 47], but few studies have been conducted on its relationship with genetic markers. Therefore, this is a new evidence to support BAD-D as an effective diagnostic indicator of keratoconus. BAD-D is a comprehensive indicator of anterior segment analysis system (Pentacam), which was calculated based on the linear regression analysis of the tomographic index (the height and thickness of the anterior and posterior surface of the cornea). CBI is a comprehensive biomechanical parameter in the dynamic corneal response parameters measured by Corvis ST. And calculating TBI through BAD-D and CBI can better distinguish potential corneal dilatation. It means that the two corneal parameters, BAD-D and CBI, have a comprehensive effect on TBI. In this study, the result of TBI may be greatly affected by CBI when analyzing their correlation with the genotypes of rs1324183 in KC, and the weak correlation between CBI and KC risk genotype (genotype AA of rs1324183) is likely to weaken the correlation between TBI and KC risk genotype strongly.

All in all, although we have observed that TBI, BAD-D, and CBI, which are the top three in diagnostic efficacy, can well distinguish KC from normal people (Table 4, Fig. 2), only BAD-D is significantly associated with high-risk genotype AA of rs1324183 of KC patients. It suggests that the diagnosis criterion of KC is complex and diverse. The published results still need to be verified widely.

In this case–control study, the KC patients with typical clinical characteristics were mainly from the completion period, with a BCVA < 0.8. And we were unable to compare the correlation between the SNP rs1324183 (*MPDZ-NFIB*) and different degrees of keratoconus for lacking of sufficient number in patients from incubation period, initial period, and scarring period. While our results showed a potential of BAD-D in predicting the risk of keratoconus, further studies in multiple populations involving different severities of keratoconus are warranted.

## Conclusions

We were able to replicate a correlation of rs1324183 (*MPDZ-NF1B*) with KC in our population. Our results further better illustrates the significance of BAD-D as a diagnostic indicator for KC, and the KC risk genotype, genotype AA of SNP rs1324183, would also probably be a reliable early diagnosis indicator in populations of China, and they both should be noted carefully in its future studies.

## Supplementary Information

**Additional file 1: Table S1.** Raw data for the control group.

**Additional file 2: Table S2.** Raw data for the KC group.

## Data Availability

All data generated or analysed during this study are included in this published article [and its supplementary information files].

## Abbreviations

KC: Keratoconus
TP: The thinnest point thickness
K max: Max keratometry
BAD-D: Belin/Ambrósio enhanced ectasia display final D value
Df: Deviation of normality of the front elevation
Db: Deviation of normality of the back elevation
Dp: Deviation of normality of pachymetric progression
Dt: Deviation of normality of corneal thinnest point
Da: Deviation of normality of relational thickness
ARTH: Ambrósio’s relational thickness
CBI: Corvis biomechanical index
Da_Ratio: Deformation amplitude ratio
SPA1: Adjusted AP1-bIOP
bIOP: Biomechanical intra occular pressure; integrated radius
TBI: Tomographic and biomechanical index
AUC: Area Under Curve
ROC: Receiver Operating Characteristics curve
OR: Odds Risk
BCVA: Best-corrected visual acuity
IOP: Intraocular pressure
